# Science for art: multi-years' evaluations of biocidal efficacy in support of artwork conservation

**DOI:** 10.3389/fmicb.2023.1178900

**Published:** 2023-06-09

**Authors:** Flavia Bartoli, Daniela Isola, Annalaura Casanova Municchia, Alma Kumbaric, Giulia Caneva

**Affiliations:** ^1^Institute of Heritage Science [Centro Nazionale delle Ricerche - Istituto di Scienze del Patrimonio Culturale (CNR-ISPC)], National Research Council of Italy, Area della Ricerca di Roma 1, Rome, Italy; ^2^Department of Sciences, University of Roma Tre, Rome, Italy; ^3^Department of Economics, Engineering, Society and Business Organization (DEIM), University of Tuscia, Largo dell'Università snc, Viterbo, Italy; ^4^National Biodiversity Future Center (NBFC), Università di Palermo, Palermo, Italy

**Keywords:** Bio-Art, science for cultural heritage, cyanobacteria, street art, treatments durability, biological recolonization, Tiber embankments colonization

## Abstract

In recent decades, the relationship between Science and Art has been gradually strengthened through the use of diagnostic, conservation, and valorization technologies. New technologies can also be used to support the creation and durability of bio-artworks. Within such a context, starting from the Spring of 2014, we performed *in situ* experimentations to eventually increase the durability of the graphical artwork of William Kentridge on the Lungotevere embankments, whose creation was scheduled in the following years. We applied various combinations and concentrations of three different biocides (Algophase, Biotin R, and Preventol R80) and two water repellents (Hydrophase surfaces and Silo 111) on 34 test areas. However, the artist preferred to leave his artwork to a natural fading. Right before the realization of the graphical artwork “*Triumph and Laments of Rome”* in 2016, just the black biological colonizations mainly composed of cyanobacteria were removed through pressurized water. We monitored the artist's work through analyses of images and colorimetric variations and such drawings showed a duration of 4 years in the natural conditions of recolonization. Here we show how the recolonization of treated and control areas, analyzed with the same methods, showed an increased duration, 3 years longer than under natural conditions in the case of Preventol R80^®^ and Biotin R^®^ plus Silo 111^®^. The tested solutions showed differential effectiveness and multiple possibilities of use to support the maintenance of the artwork if the artist wanted to preserve his artwork for a longer period.

## 1. Introduction

Science and scientists can support artists who want to realize artworks with natural and living materials. In fact, in recent decades, the relationship between Science and Art has been gradually strengthened by applying technologies for diagnostics, conservation, and the realization of artworks. Indeed, the scientific support offered to artists in creating and maintaining artworks using elements of nature is increasingly spreading (Kac, 2006; Kallergi, 2008; Idema, 2012; Yetisen et al., 2015; Gemtou, 2021). These contemporary artistic currents that concern the creation of artworks operating with biological growths are based on a variety of interdisciplinary approaches between Art and Science (Kac, 2006; Stracey, 2009; Idema, 2012; Anker, 2014; Sharma, 2014; Gemtou, 2021).

This type of artistic representation is quite recent. However, the first bio-artwork exposition was in 1933 when Alexander Fleming, who discovered the first antibiotic (i.e., penicillin), showed in a hospital aisle his “bacterial paintings”, obtained by inoculating bacteria on paper previously imbibed by culture medium (Stracey, 2009). The 1990s saw a more recent diffusion of this kind of art; for example, the Japanese artist Jun Takita became famous with his moss bioluminescence project, while the artist Diego Scroppo produced drawings using the bioluminescent fungus *Panellus stipticus* (Pascucci et al., 2016). Bio-Art has multiple possible applications, for instance the competition *Agar Art*, which was organized in 2015 by the American Society for Microbiology to draw attention to the world of microorganisms, showing their potential use for creating artistic paintings. Similarly, we cite *The Urban Biome Map*, 2^nd^ place in the 2015 ASM Agar Art Contest, a multidisciplinary project involving citizens, scientists, and artists, aiming to show the personalized microbiome of the citizens of New York (ASM Agar Art Contest, 2015, ASM.org).

These kinds of projects in a natural environment are rare. However, in Rome, particularly, the Tiber River Embankments have been the canvas for two living artworks. Such riverbank walls, built between 1875 and 1926 to protect the city from flooding, are an important part of Rome's urban history developed over about 8 km and with a height of 18 m. They have a constant slope (80°) and are exclusively composed of travertine slabs from the outcrops of the *Acque Albule* thermal complex of the Tiburtini Mountains near Tivoli (Rome). The homogeneous substrate, its high micro- and macroporosity, and its inclination highly influence the amount of water retained and the bioreceptivity of the stone (Caneva et al., 2004). These characteristics are fundamental to explaining the biological origin of their surfaces' diffuse and homogeneous blackening (Bellinzoni et al., 2003; Kumbaric et al., 2012; Antonelli et al., 2020). Previous studies have characterized the communities of microflora that were composed mainly of cyanobacteria and green algae (Bartoli et al., 2021a). Considering the bioclimatic conditions of Rome, and the xeric conditions of the walls' embankments, exposed directly to high sunlight, the dominant organisms resulted in cyanobacteria, as commonly shown in similar conditions (Guillitte, 1995; Caneva et al., 2005, 2016; Barberousse et al., 2006). The taxonomic investigations showed that the walls are colonized by cyanobacteria such as *Chroococcus lithophilus* Erceg., *Myxosarcina spectabilis* Geitler, *Tolypothrix byssoidea* Kirch., *Synechocistis pevalekii* Erceg., *Gloeocapsa biformis* Erceg., *Nostoc punctiforme* Hariot, *Synechococcus aeruginosus* Nägeli, *Scytonema julianum* Meneghini ex B.A.Whitton and to a less extent green algae *Desmococcus olivaceus* (Persoon ex Acharius) J.R.Laundon, *Muriella terrestris* J.B.Petersen, *Chlorococcum* sp., and black meristematic fungi (Bellinzoni et al., 2003). Further bacteria were identified through biomolecular analysis (Antonelli et al., 2020).

Using this biological blackening spread on the Tiber River Embankments, in 2005, the American artist Kristin Johns, with her project “*She-Wolves”*, realized twelve figures as an iconic symbol of the foundation of Rome. The drawings were obtained by removing the biological patina through a pressure washer, forming the final figure as a sort of negative. The artwork lasted a few years due to the natural recolonization, but a detailed assessment of their duration was not carried out. Following the requests of Kristin Johns, starting in the Spring of 2014, we performed *in situ* experimentation in support of further artistic realizations, testing different mixtures of water repellents and biocides to delay the artworks' fading and loss.

Later, thanks to Kristin Jones' support through the foundation of TEVERETERNO, in 2016, the South African artist William Kentridge developed his project “*Triumphs and Laments”*, a procession of eighty figures which describe glorious and sad episodes of the history of the Eternal City, along the embankments between Ponte Sisto and Ponte Giuseppe Mazzini. In this case, Kentridge preferred to leave his artwork to fade naturally. Our previous monitoring of this artwork, analyzing image and colorimetric variations of the black patinas, showed a duration of 4 years in natural conditions of recolonization (from 2016–2020, Bartoli et al., 2021a).

In conservation science, biocides and water repellents are well known as direct treatments of biodeteriogens to avoid or delay natural colonization. Mainly, studies focus on laboratory tests (Nugari et al., 1993; Tiano et al., 1994; Bartolini et al., 2007; Urzí and De Leo, 2007; Moreau et al., 2008; Delgado and Charola, 2011; Pinna et al., 2012; Pinna, 2017; Jeong et al., 2018; Toreno et al., 2018; Fidanza and Caneva, 2019; Bartoli et al., 2021b) and are performed on a short-medium time scale (less than 3 years). However, *in situ*, the experimental conditions cannot be controlled. The studies on a long-time scale (multiyear, between 3–12 years) are scarce in the literature, given the complexity of execution (Charola et al., 2007; Nascimbene et al., 2009; Delgado Rodrigues et al., 2011; Salvadori and Charola, 2011; Pinna et al., 2018; Caneva et al., 2019). Considering the lack of research on technologies and techniques applied to the support of bio-artwork realization and the paucity of *in situ* long-timescale monitoring, this study aims to understand which chemical treatments could delay biological growth in a cleaned area, extending the lifetime of images and the dynamic recolonization process in different test conditions.

## 2. Materials and methods

**Figure 1:**
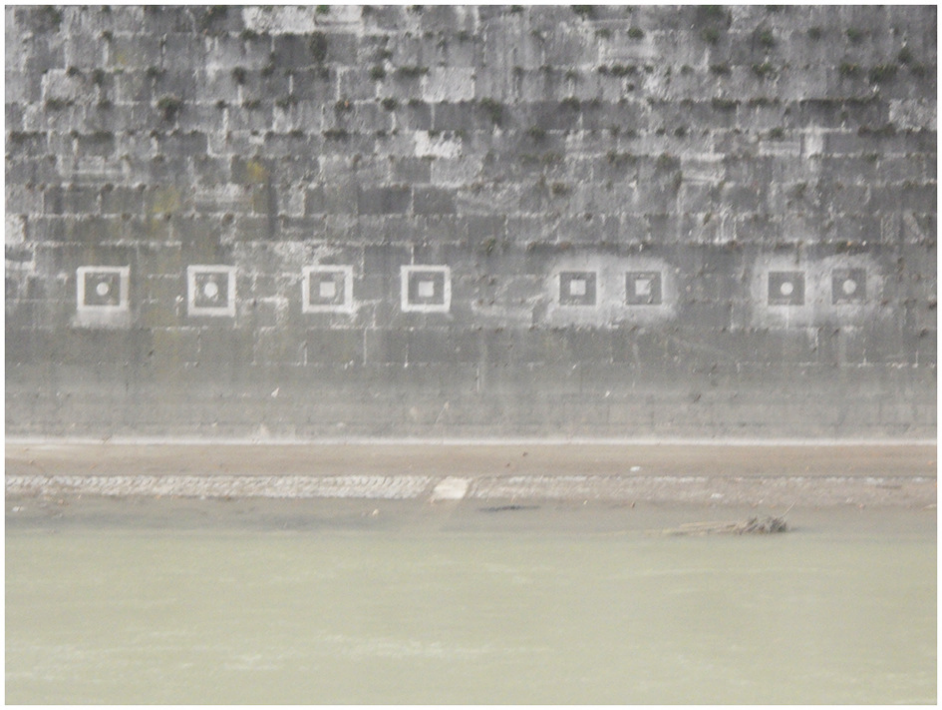
Test areas on Tiber embankments near to Regina Margherita Bridge.

A stretch of the Lungotevere near the Regina Margherita Bridge was selected as a test area. In October 2014, eight squares of the embarkment with an overall size of 64 × 64 cm were treated with the same techniques that were used by the artist while making the friezes, including white and gray areas, to simulate the nuances used in the drawings (Figure 1).

According to literature data on the toxicity and the safety for users and the environment (Caneva et al., 2008; Pascucci et al., 2016), and considering the customer's request, we selected three different biocides (Preventol ^®^R80, Algophase^®^, and Biotin R^®^) and two water-repellents (Silo 111^®^ and Hydrophase Superfici^®^). We chose mixtures of biocides and water repellents according to previous data on their effectiveness in preventing biological colonization (Charola et al., 2007; Urzí and De Leo, 2007; Pinna et al., 2012), as reported in Table 1.

**Table 1:** Selected biocides and water repellents and their concentrations and mixtures.

**Biocide**	**Active principle**	**Mixture and concentration**	**Tests**	**Mixture and application**
Preventol R80^®^	alkyl-dimethyl-benzylamine chloride	4% in distilled water	T1', T1”, T1”' T2', T2”, T2”' T3', T3”, T3”' T4', T4”, T4”' T5', T5”, T5”' T6', T6”, T6”' T7', T7”, T7”' T8', T8”, T8”' T9', T9”, T9”' T10', T10”, T10”' C', C”, C”', C””	PR80 + Alg + Hydro-5% PR80 + Alg + Hydro-3% PR80 + BTr + Silo111-4% PR80 + BTr + Silo111-3% Alg + Hydro-5% Alg + Hydro-3% BTr + Silo111-4% BTr + Silo111-3% Silo111 10% Hydro 40% Control
Algophase^®^	2,3,5,6-tetrachloro-4-methylsulfonyl- pyridine	5–3% of Algophase directly in Hydrophase Superfici		
Biotin R^®^	OIT and Carbamate	4–3% of Biotin R directly in silo 111		
**Water repellents**	**Active principle**	**Concentration**		
Silo 111^®^	Methylethoxy polysiloxane	10% in white spirit		
Hydrophase Superfici^®^	Alkyl alcoxy silane	40% in isopropyl alcohol		

Preventol ^®^R80, widely used in restoration, can induce interference problems causing color variations on the substrate (Nugari et al., 1993). It has been shown to have a slight algaecide power at low concentrations (1%). We deemed it safer to use Preventol ^®^R80 at a low concentration, in combination with the other two biocides (Algophase^®^, Biotin R^®^).

We selected ten different dowels (10.5 × 4 cm each) as single test areas for the mixtures and concentrations applied in three series of repetitions (', ”, ”'), for a total of thirty dowels, with the scheme defined in Table 1. We also monitored the natural recolonization of the control untreated test areas (C). We applied all products with a paintbrush. Four test areas (T1, 2, 3, and 4) were pre-treated with Preventol R80 alone, before applying the biocide-water-repellent mixture to evaluate the differences without pre-treatment (T5, 6, 7, and 8). Moreover, we tested how it was possible to obtain nuances for the next eventual frieze. They would be created by reducing the cleaning of the surface and creating gray areas in which the colonization was not completely removed. In these areas, we could not apply biocides, as we would risk killing the microorganisms left to obtain the gray shades. Therefore, we applied water repellents on some gray tiles (T9 and T10) to see if we could delay the colonization to some extent.

In April 2014, 2 months after the product application, we began monitoring the test areas every 2 months until February 2015. Considering the few differences observed with the naked eye, we reduced the frequency of monitoring visits to one time *per* year until October 2022, when we observed the total disappearance of the test areas. The monitoring protocol is described below.

In October 2022, we collected biological samples to understand if the chemical-physical treatments affected the general composition of the communities. We selected the dowels without any treatment (BO = original black) and with only the physical removal of the biological patina (BA = black after cleaning, control test). We also selected the dowels that were pre-treated with Preventol R80 and those treated with the minimum and maximum products' application (T1', T1”' and T3', T3”'). We took sterile biological samples using a scalpel and we analyzed the fresh samples under an optical microscope (Olympus BX41) at different magnifications.

We performed field monitoring by evaluating the color changes with an EOPTIS CLM-194 handheld colorimeter with a measurement diameter of 8 mm according to the standard procedures defined in the European Standard EN15886:2010. We took ten measurements for each test area, trying to avoid the most significant holes and to minimize the influence of the superficial porosity of the travertine colorimetric measures (Caneva et al., 2004). After, we calculated the average values for the results obtained on the test areas that were treated in the same way to have a single data point.

To quantify the color variations, we used the color variation values recognized by the CIE-L^*^a^*^b^*^ system (UNI-EN-15886, 2010). The color coordinates L^*^, a^*^, and b^*^ determine the color location in the CIELAB color space: L^*^ indicates lightness, a^*^ redness– greenness, and b^*^ (yellowness–blueness). We measured L^*^, a^*^, and b^*^ values in selected treated areas (T1-T10) and in four untreated control test areas (C). Then, we calculated the total color variation (ΔE^*^) from three color parameters with the formula: ΔE = [(ΔL^*^)^2^ + (Δa^*^)^2^ + (Δb^*^)^2^]^1/2^. The variations of the parameters L^*^, a^*^, and b^*^ were calculated by evaluating the distance between the data points on each treated test area during the seven years of monitoring and the date of the first measurements, acquired on the single test areas in April 2014 (T_0_) (i.e., L^*^ treated – L^*^ T_0_). An increase in ΔE^*^ and a decrease in ΔL^*^ values can be related to recolonization processes. This phenomenon can be observed when the treated surface becomes darker and greener (decrease in Δa^*^) due to the growth of photosynthetic microorganisms. The values of color variation ΔE^*^ over the threshold value of four were considered according to the literature (Pinna, 2017, 2022; Eyssautier-Chuine et al., 2018; Toreno et al., 2018) and our previous work on the Tiber embankments (Bartoli et al., 2021a). Indeed, restorers, and conservators defined this value as the limit where the human eye can see the total color change, so it was used to define the resumption of biological colonization. Lightness value results indicate a darkening when ΔL^*^ values become negative. The data have been compared with the results obtained from the dowels that were not treated and thus represent natural colonization. The comparison between the treated and untreated areas was impossible to do in the same area because the control areas were subject to natural colonization.

For this reason, we have compared our results with the first measurements made at the beginning. As reported in the literature (Vergès-Belmin et al., 2008), the critical aspect in taking color measurements outdoors is due to different humidity conditions because the substrate contains different amounts of water. So, different water contents could influence the final colorimetric data. To minimize this problem, we have preemptively checked the micro climatic conditions to choose more similar ones, avoiding critical situations such as rainy periods.

At last, we collected climatic data of the sampling days (i.e., precipitation and average temperature) to choose the best periods to compare the colorimetric data in order to determine whether the differences of the colorimetric measurements were linked to weather conditions of the sampling days or to changes in the blackening on the surface. For this reason, we have compared the colorimetric measures performed in April/May from 2014 until 2021 and the first measure in October 2014 with the results obtained during the last survey in October 2021. Each colorimetric measure was taken during days and periods without rain.

## 3. Results

### 3.1. Microscopical observation of the biological patina

**Figure 2:**
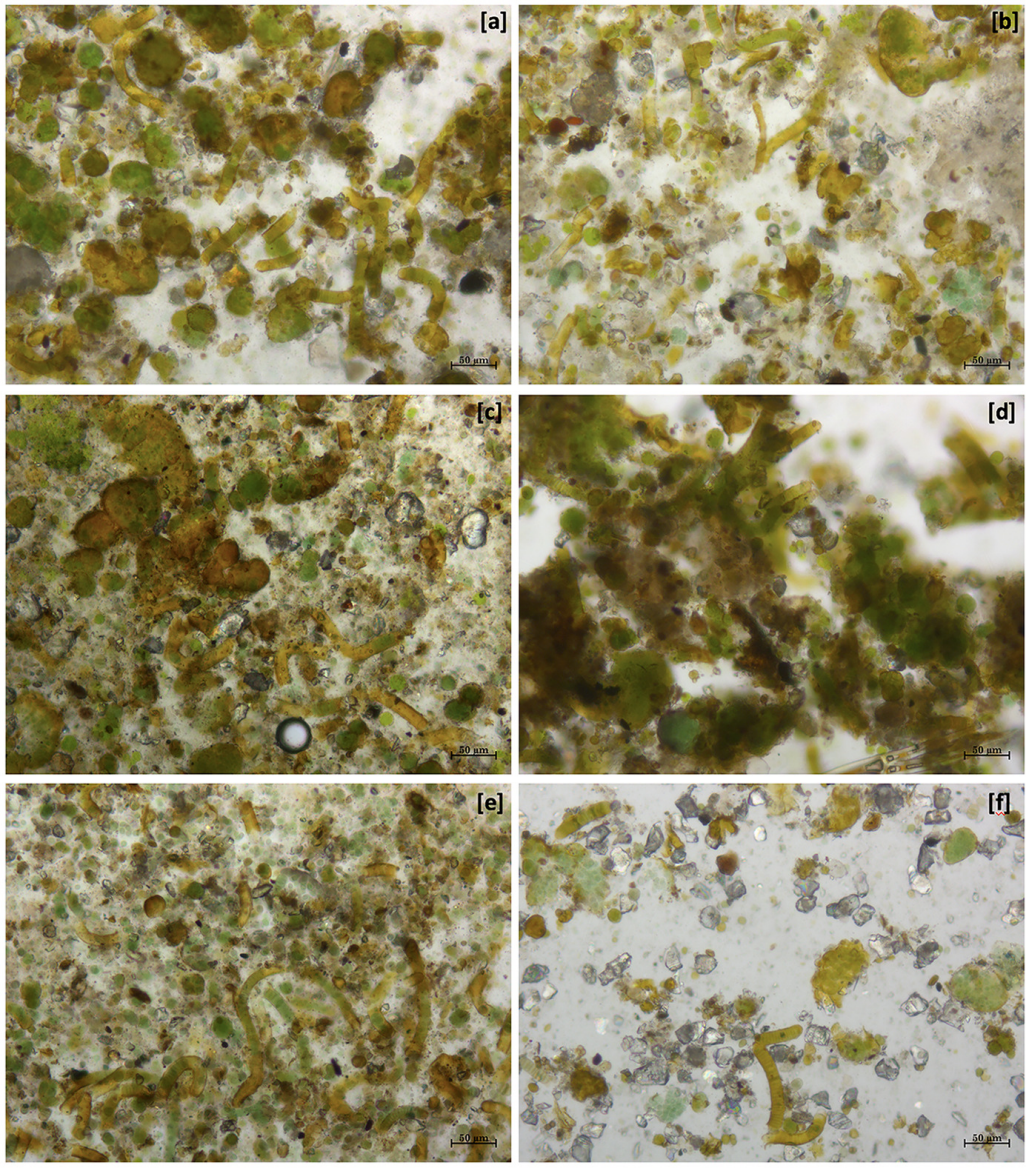
Optical image at 10X magnification of different dowels on 22 October 2022. **(a)** BO, the community without any kind of alteration; **(b)** BA, recolonizing communities after only physical cleaning; **(c)** T1' dowel, recolonizing community after 1 application of PR80 + Alg + Hydro-5% and **(d)** T1”', with three applications; **(e)** T3' dowel, recolonizing community after 1 application of PR80 + BTr + Silo-4% and **(f)** T3”', with three applications.

The comparison between the communities collected in the untreated and treated areas with the maximum concentration of biocide-hydro repellent solutions showed only a few differences in the species composition (Figure 2). The observation of the fresh preparations under an optical microscope at several magnifications highlighted that there was an evident difference in abundance of microorganisms during the recolonization phase.

The comparison between BO and BA (Figures 2a, b) showed the same composition, but in the second one, there is a smaller colonization, such as in the other comparison T1' vs. T1”' and T3' vs. T3”'. Moreover, the microscopic analysis showed that the three applications of PR80 + BTr + Silo-4% determined a considerable reduction of the recolonization rate (Figure 2f) but also a few changes in the biological colonization such as the absence of algal components in the community.

### 3.2. Application of different treatments

**Figure 3:**
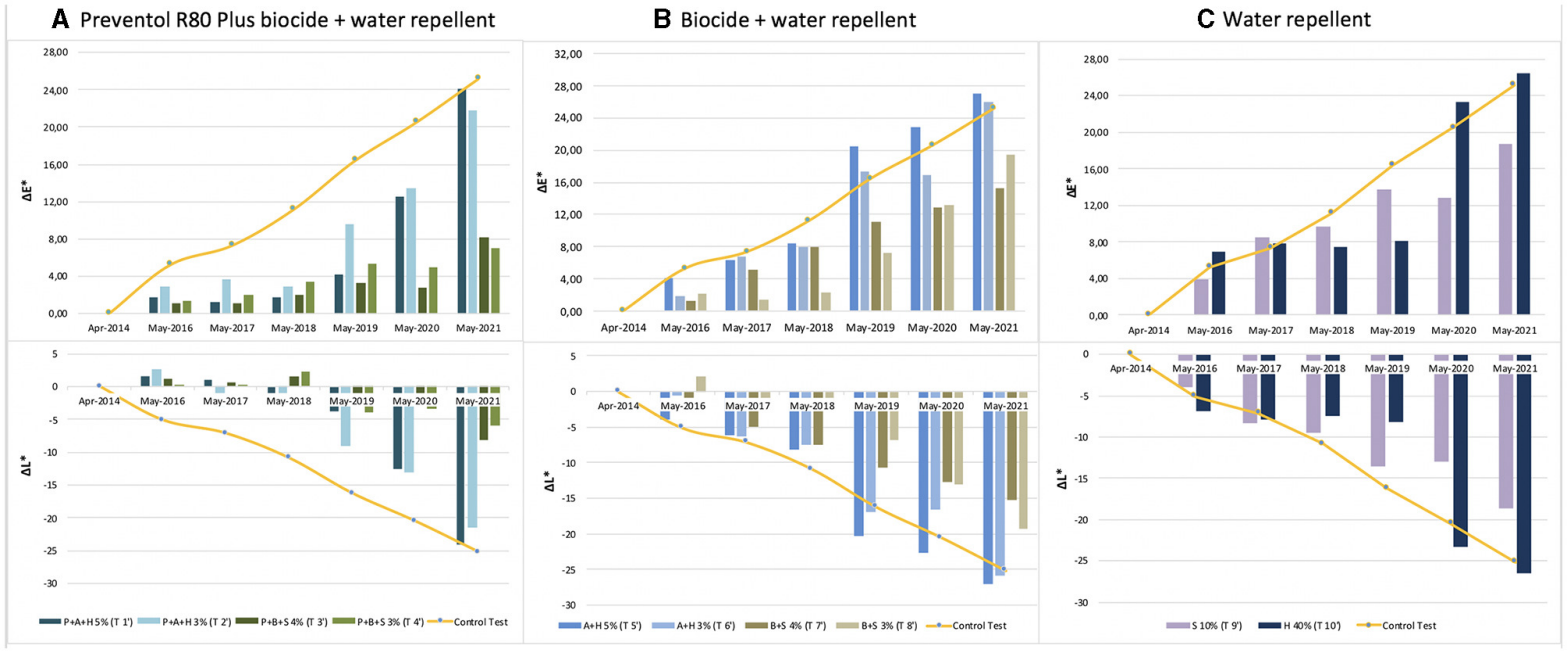
Color and lightness variation (ΔE* and ΔL*) in the test areas treated with a single biocide application: **(A)** Preventol treatment followed by a mix of biocide and hydro repellent; **(B)** mix of biocide and hydro repellent; **(C)** only hydro repellent application. P, Preventol; A, Algophase; H, Hydrophase; B, Biotin R; S, Silo 111.

The color variation values ΔE^*^ over the threshold value of four, as shown in Figure 3 and Table 2, were reached after 5 years (2019) with the application (repeated once) of all products, except for the product containing Preventol + Biotin R + Silo 111 with a 4% concentration. In May 2021, all test areas were completely recolonized (Figure 3). However, the recolonization is more evident in those areas treated with Preventol + Algophase + Hydrophase at both concentrations (5 and 3%), where the trend of ΔE ^*^data was similar to the data of the control test area that was not treated. Although the areas treated with Preventol + Biotin R + Silo 111 at both concentrations (5 and 3%) also greatly exceed the threshold ΔE value of four, they still appear to have a lower coverage than the areas treated with other biocide mixtures and the coverage of the control test areas.

**Table 2:** Colorimetric results regarding the area treated by Preventol plus biocide mixed with hydrorepellent and the control test area from the starting point (April 2014).

			**3/4/2014**	**16/10/2014**	**17/2/2015**	**9/5/2016**	**30/5/2017**	**22/5/2018**	**24/5/2019**	**26/5/2020**	**20/5/2021**	**18/10/2022**
Preventol plus biocide mixed with hydrorepellent	T1'	ΔL	0.00	2.91	1.60	1.64	1.14	−1.73	−10.56	−12.53	−24.08	−17.14
		Δa	0.00	0.49	0.48	0.30	0.31	0.24	−0.18	−0.82	−1.25	−1.51
		Δb	0.00	6.74	1.11	0.59	0.39	−0.01	−1.05	−0.80	−1.52	−0.78
		ΔE	0.00	7.36	2.00	1.77	1.25	1.75	10.61	12.58	24.16	17.22
	T1”	ΔL	0.00	2.81	1.31	−1.15	−0.55	−0.61	−7.95	−2.62	−10.77	−15.44
		Δa	0.00	0.10	−0.13	−0.31	−0.43	−0.48	−0.90	−1.41	−1.96	−2.19
		Δb	0.00	0.40	0.28	−0.90	−1.87	−1.27	−2.90	−2.00	−1.63	−1.83
		ΔE	0.00	2.84	1.35	1.49	2.00	1.49	8.51	3.58	11.07	15.70
	T1”'	ΔL	0.00	2.68	2.40	4.17	1.91	−0.83	−7.78	−6.63	−19.70	−17.56
		Δa	0.00	0.32	0.17	0.18	0.08	0.02	−0.13	−0.49	−1.35	−1.42
		Δb	0.00	1.91	1.44	1.51	0.80	0.72	−0.61	0.38	0.82	0.55
		ΔE	0.00	3.31	2.80	4.43	2.07	1.10	7.80	6.66	19.77	17.63
	T2'	ΔL	0.00	0.40	−0.97	2.60	−3.01	−1.70	−9.00	−13.05	−21.53	−17.42
		Δa	0.00	−0.14	−0.42	−0.28	−0.64	−0.64	−0.78	−1.56	−2.33	−2.37
		Δb	0.00	−0.67	−1.09	−1.37	−2.10	−2.24	−3.35	−2.59	−2.71	−2.81
		ΔE	0.00	0.79	1.51	2.95	3.73	2.89	9.63	13.39	21.83	17.80
	T2”	ΔL	0.00	0.82	−0.36	−0.44	−5.31	−7.46	−11.83	−13.57	−21.21	−22.65
		Δa	0.00	0.25	0.05	−0.09	−0.35	−0.55	−0.70	−1.18	−1.96	−1.69
		Δb	0.00	0.87	0.37	−0.49	−1.47	−1.82	−2.13	−2.36	−2.44	−1.90
		ΔE	0.00	1.22	0.52	0.66	5.52	7.70	12.04	13.83	21.44	22.79
	T2”'	ΔL	0.00	0.98	−0.73	−1.15	−4.48	−2.17	−16.73	−17.10	−33.39	−29.35
		Δa	0.00	0.11	−0.17	−0.18	−0.32	−0.39	−0.47	−0.78	−1.57	−1.22
		Δb	0.00	−0.07	−0.11	−0.62	−0.79	−1.09	−2.32	−1.65	−1.59	−0.90
		ΔE	0.00	0.99	0.76	1.32	4.56	2.46	16.90	17.19	33.46	29.39
	T3'	ΔL	0.00	2.72	1.72	1.16	0.65	1.55	−2.75	−2.62	−8.10	−16.92
		Δa	0.00	−0.01	−0.07	−0.09	−0.33	−0.36	−0.53	−0.42	−1.35	−1.88
		Δb	0.00	−0.40	0.35	−0.04	−0.91	−1.15	−1.78	−0.68	−0.70	−2.17
		ΔE	0.00	2.75	1.76	1.16	1.17	1.97	3.32	2.74	8.24	17.16
	T3”	ΔL	0.00	4.25	2.19	1.53	3.95	2.27	−0.68	−6.89	−11.02	−15.74
		Δa	0.00	−0.10	−0.21	−0.21	−0.32	−0.03	−0.54	−1.83	−2.48	−2.03
		Δb	0.00	−0.40	0.07	0.05	−0.57	−0.30	−1.24	−2.20	−1.92	−2.16
		ΔE	0.00	4.27	2.20	1.54	4.01	2.29	1.51	7.46	11.46	16.02
	T3”'	ΔL	0.00	5.78	2.69	4.84	3.37	5.74	−0.86	−9.81	−7.09	−6.27
		Δa	0.00	0.02	−0.27	−0.30	−0.36	−0.42	−0.52	−1.05	−1.74	−1.70
		Δb	0.00	0.72	1.00	1.12	0.36	0.44	−0.60	−1.68	0.58	0.52
		ΔE	0.00	5.83	2.88	4.98	3.41	5.77	1.17	10.01	7.32	6.51
	T4'	ΔL	0.00	2.53	1.20	0.33	0.11	2.30	−3.93	−3.32	−5.91	−11.32
		Δa	0.00	−0.29	−0.42	−0.53	−0.73	−0.83	−1.10	−1.94	−2.66	−2.43
		Δb	0.00	−1.37	−0.97	−1.28	−1.86	−2.45	−3.39	−3.14	−2.82	−3.32
		ΔE	0.00	2.89	1.59	1.43	2.01	3.46	5.31	4.96	7.07	12.05
	T4”	ΔL	0.00	1.72	−0.21	−0.08	−1.30	−3.14	−6.71	−13.68	−22.85	−14.36
		Δa	0.00	0.02	−0.19	−0.01	−0.08	−0.26	−0.41	−0.45	−1.51	−1.71
		Δb	0.00	0.24	0.06	1.23	0.78	0.14	−0.46	−0.17	−0.85	0.91
		ΔE	0.00	1.74	0.29	1.23	1.52	3.15	6.74	13.69	22.91	14.49
	T4”'	ΔL	0.00	0.70	−0.85	−0.06	−3.16	−1.43	−8.11	−6.73	−19.19	−16.41
		Δa	0.00	−0.10	−0.24	−0.17	−0.24	−0.36	−0.52	−0.89	−1.71	−1.79
		Δb	0.00	−0.74	0.05	−0.22	−0.69	−1.09	−1.75	−1.13	−1.65	−1.05
		ΔE	0.00	1.02	0.88	0.28	3.24	1.83	8.31	6.89	19.33	16.54
Control test	C	ΔL	0.00	−0.63	−3.35	−5.10	−7.13	−10.87	−16.22	−20.48	−25.14	−26.36
		Δa	0.00	0.14	−0.06	−0.11	−0.25	−0.33	−0.46	−1.19	−1.33	−1.41
		Δb	0.00	−0.26	−0.36	−1.26	−1.65	−2.60	−2.56	−1.42	−0.82	−1.33
		ΔE	0.00	0.69	3.37	5.26	7.33	11.19	16.43	20.56	25.19	26.43

**Figure 4:**
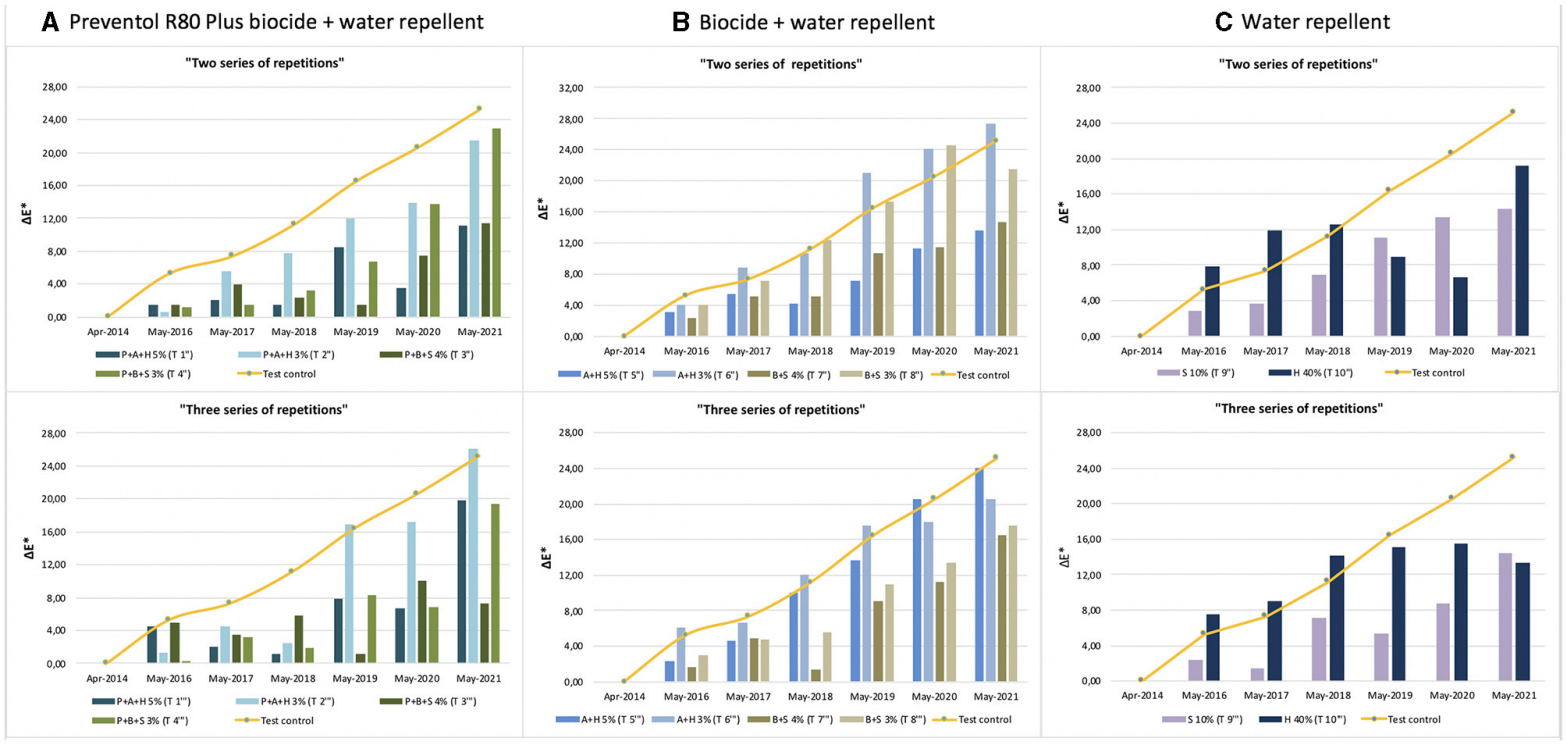
Color variation (ΔE*) in the test areas treated with two and three series of repetitions: **(A)** Preventol treatment followed by a mix of biocide and hydro repellent; **(B)** mix of biocide and hydro repellent; **(C)** only hydro repellent application. P, Preventol; A, Algophase; H, Hydrophase; B, Biotin R; S, Silo 111.

**Figure 5:**
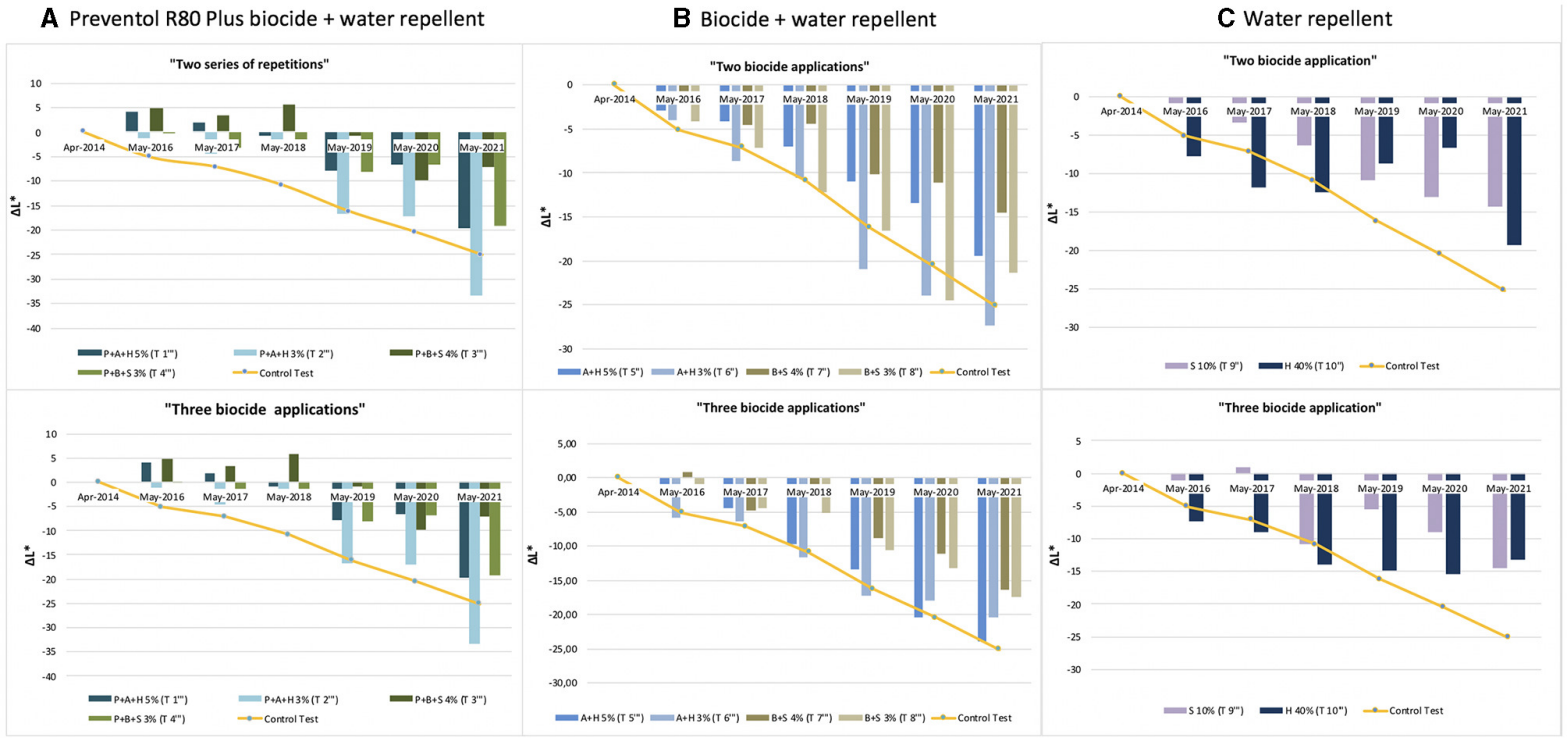
Lightness variation (ΔL*) in the test areas treated with two and three series of repetitions: **(A)** Preventol treatment followed by a mix of biocide and hydro repellent; **(B)** mix of biocide and hydro repellent; **(C)** only hydro repellent application. P, Preventol; A, Algophase; H, Hydrophase; B, Biotin R; S, Silo 111.

Furthermore, based on the results, the additional re-application of biocides has not affected the colonization delay. In fact, all four biocides in 2021 exceed the ΔE ^*^ threshold (Figures 4, 5). In some cases, such as with the Preventol + Biotin R + Silo 111 with a 3% concentration, the results seemed worse, showing a more significant color variation than with a single application. In May 2016, 2 years after the first treatment, all the areas treated only once showed no darkening, unlike the untreated control areas where negative values were recorded (Figure 3). In May 2017, the surfaces treated with the Preventol + Algophase + Hydrophase 3% product began to darken, while the surfaces treated with the same product at a concentration of 5% began to darken the following year. In May 2019, all the treated areas started to darken (Figure 3). Starting from May 2021, the areas treated with Preventol + Algophase + Hydrophase at both concentrations have shown the same trend as untreated control areas. The lightness results showed that tiles in which the products were applied multiple times (twice or thrice) have shown no improvement in the overall data (Figures 4, 5). On the contrary, the area treated with Preventol + Algophase + Hydrophase 3% has shown a darkening since 2016. Furthermore, in 2021 the tiles treated three times with this product seemed to perform worse than the untreated control areas (Figure 5). Table 2 also shows a decrease in Δa^*^ values during the 7 years, showing a greenish tone that confirms a recolonization phenomenon. In October 2022, we observed that all four biocides had slowed down the recolonization with less blackening than the control test (ΔE < 25).

The application of only the mixture with the biocide and water repellent without the preventive application of Preventol showed a test behavior like the natural situation recorded on the control tiles without the application of the products. Since 2017, three products (Algophase + Hydrophase 3 and 5%, and Biotin R and Silo 111 4%) out of four, applied once, had already exceeded the threshold ΔE^*^ value of four CIELAB units, showing a visible recolonization of the surface (Figure 3, Table 3). In 2019, all the treated areas with the four products appeared visible (ΔE ^*^> 4). Test areas treated with Algophase + Hydrophase 3 and 5% followed the similar trend of the untreated areas of the control test (Figure 3). The application of higher amounts of biocides has not slowed down the colonization. Figures 4, 5 show that since 2017 all the treated areas have darkened. Since 2017, the areas treated with products containing Algophase + Hydrophase have had the same trend as the untreated control areas. The repetitive application of biocides repetitions did not seem to influence the colonization behavior. In fact, in 2021, a negative lightness value ΔL ^*^= ~-25 was achieved in one, two, and three re-applications of the product (Figures 4, 5). The decrease in Δa^*^ values points out a potential presence of photosynthetic microorganisms that increased at the end of the seven years (Table 3). In October 2022, the dowels were no longer visible, so we could not monitor them.

**Table 3:** Colorimetric results regarding the area treated by biocide mixed with hydro repellent and the control test area, from the starting point (April 2014).

			**3/4/2014**	**16/10/2014**	**17/2/2015**	**9/5/2016**	**30/5/2017**	**22/5/2018**	**24/5/2019**	**26/5/2020**	**20/5/2021**	**18/10/2022**
Biocide mixed with hydrorepellent	T5'	ΔL	0.00	1.02	0.53	−3.89	−6.12	−8.11	−20.29	−22.72	−27.01	0.00
		Δa	0.00	0.49	0.35	0.36	0.19	0.29	0.15	−0.07	−0.59	0.00
		Δb	0.00	−0.06	0.22	−0.97	−1.49	−2.21	−3.24	−2.29	−2.06	0.00
		ΔE	0.00	1.14	0.67	4.03	6.30	8.41	20.54	22.84	27.10	0.00
	T5”	ΔL	0.00	1.80	−1.27	−2.89	−5.41	−4.12	−6.95	−10.99	−13.46	−19.49
		Δa	0.00	0.31	0.26	0.02	0.01	0.04	−0.20	−0.31	−0.88	−1.51
		Δb	0.00	1.05	0.43	−1.13	−0.74	−1.02	−1.83	−2.35	−1.74	−1.97
		ΔE	0.00	2.11	1.37	3.10	5.46	4.24	7.19	11.24	13.60	19.65
	T5”'	ΔL	0.00	−0.65	1.57	−1.92	−4.41	−9.67	−13.37	−20.42	−23.99	^*^
		Δa	0.00	0.45	0.20	0.30	0.09	0.01	0.20	−0.15	−0.68	^*^
		Δb	0.00	−0.28	0.05	−1.16	−1.50	−2.55	−2.67	−1.93	−1.97	^*^
		ΔE	0.00	0.84	1.58	2.26	4.66	10.00	13.64	20.51	24.08	^*^
	T6'	ΔL	0.00	0.99	−0.90	−0.61	−6.35	−7.51	−16.96	−16.65	−25.79	^*^
		Δa	0.00	0.15	−0.02	−0.17	−0.15	−0.25	−0.42	−0.69	−1.26	^*^
		Δb	0.00	−0.54	−0.23	−1.67	−2.36	−2.71	−3.73	−2.64	−2.40	^*^
		ΔE	0.00	1.14	0.93	1.79	6.78	7.99	17.37	16.87	25.93	^*^
	T6”	ΔL	0.00	−0.90	−4.17	−4.04	−8.71	−10.58	−20.93	−23.99	−33.97	^*^
		Δa	0.00	0.30	0.23	0.21	0.34	0.13	−0.02	−0.48	−0.62	^*^
		Δb	0.00	0.91	0.60	−0.21	−1.33	−1.90	−2.61	−1.66	−1.30	^*^
		ΔE	0.00	1.31	4.22	4.05	8.82	10.75	21.09	24.05	34.00	^*^
	T6”'	ΔL	0.00	0.16	−4.13	−5.80	−6.32	−11.66	−17.26	−17.90	−20.44	^*^
		Δa	0.00	0.08	−0.07	−0.01	−0.14	−0.24	0.00	−0.33	−0.97	^*^
		Δb	0.00	−0.48	−0.46	−1.66	−2.02	−2.80	−3.17	−2.20	−1.84	^*^
		ΔE	0.00	0.51	4.16	6.03	6.64	11.99	17.55	18.04	20.54	^*^
	T7'	ΔL	0.00	5.82	3.89	−0.95	−4.96	−7.55	−10.72	−12.69	−15.24	^*^
		Δa	0.00	0.17	0.05	−0.01	−0.16	−0.32	−0.18	−0.62	−0.93	^*^
		Δb	0.00	−0.33	0.09	−0.70	−1.50	−2.48	−2.78	−1.91	−0.91	^*^
		ΔE	0.00	5.83	3.89	1.18	5.18	7.95	11.08	12.85	15.30	^*^
	T7”	ΔL	0.00	1.36	0.59	−1.85	−4.51	−4.43	−10.12	−11.12	−14.47	^*^
		Δa	0.00	−0.06	−0.23	−0.31	−0.55	−0.55	−0.59	−1.09	−1.85	^*^
		Δb	0.00	−1.14	−0.44	−1.36	−2.43	−2.55	−3.36	−2.56	−2.22	^*^
		ΔE	0.00	1.77	0.77	2.31	5.15	5.14	10.68	11.46	14.76	^*^
	T7”'	ΔL	0.00	0.81	−1.39	0.86	−4.86	−2.00	−8.91	−11.15	−16.38	^*^
		Δa	0.00	0.31	0.02	0.35	0.06	0.05	0.01	−0.53	−1.17	^*^
		Δb	0.00	−0.77	−0.20	1.34	−0.75	−0.36	−1.71	−1.54	−1.59	^*^
		ΔE	0.00	1.17	1.40	1.63	4.92	2.03	9.07	11.27	16.49	^*^
	T8'	ΔL	0.00	2.40	1.78	2.13	−1.28	−1.95	−6.86	−12.98	−19.36	^*^
		Δa	0.00	0.17	0.15	0.05	0.00	−0.19	−0.15	−0.59	−1.32	^*^
		Δb	0.00	−0.68	0.30	0.37	−0.51	−1.21	−2.26	−1.99	−1.79	^*^
		ΔE	0.00	2.50	1.81	2.16	1.38	2.30	7.22	13.15	19.49	^*^
	T8”	ΔL	0.00	−0.05	−3.01	−4.09	−7.09	−12.21	−16.65	−24.44	−21.41	^*^
		Δa	0.00	0.07	0.19	0.18	−0.06	−0.28	−0.04	−0.43	−0.97	^*^
		Δb	0.00	−0.66	0.10	−0.11	−0.65	−2.44	−2.92	−2.89	−0.57	^*^
		ΔE	0.00	0.67	3.01	4.10	7.12	12.45	16.90	24.62	21.44	^*^
	T8”'	ΔL	0.00	2.83	1.92	−2.76	−4.50	−5.13	−10.50	−13.19	−17.37	^*^
		Δa	0.00	0.03	−0.18	−0.10	−0.18	−0.28	−0.20	−0.66	−1.09	^*^
		Δb	0.00	−0.71	−0.64	−0.89	−1.35	−2.11	−2.80	−2.10	−2.01	^*^
		ΔE	0.00	2.92	2.03	2.90	4.70	5.55	10.87	13.37	17.52	^*^
Control test	C	ΔL	0.00	−0.63	−3.35	−5.10	−7.13	−10.87	−16.22	−20.48	−25.14	−26.36
		Δa	0.00	0.14	−0.06	−0.11	−0.25	−0.33	−0.46	−1.19	−1.33	−1.41
		Δb	0.00	−0.26	−0.36	−1.26	−1.65	−2.60	−2.56	−1.42	−0.82	−1.33
		ΔE	0.00	0.69	3.37	5.26	7.33	11.19	16.43	20.56	25.19	26.43

^*^Indicates the area where it was not possible to distinguish the test area.

Since 2016, a single application of the hydrorepellent has shown an increase in the colorimetric variation ΔE^*^ (Figure 3 and Table 4). In 2020 and 2021, the areas treated with Hydrophase had values comparable to those obtained in the control areas, underlining the ineffectiveness of the product in slowing down the colonization. The data about lightness values confirmed the darkening of the surfaces treated since 2016 (Figure 3). The decrease in Δa^*^ values (on average, in 2021, ~ −0.9) confirmed the presence of photosynthetic microorganisms that increased toward the end of the seven years (Table 4). An increase in the amount of the product with Silo 111, applied twice or three times, slowed down the recolonization. In fact, Figures 4, 5 showed a divergence of the data from the data of the control areas. However, as shown in Table 5, we registered major standard deviation values due to an extremely heterogeneous substrate in some test areas.

**Table 4:** Colorimetric results regarding the area treated by hydro repellent and the control test area, from the starting point (April 2014).

			**3/4/2014**	**16/10/2014**	**17/2/2015**	**9/5/2016**	**30/5/2017**	**22/5/2018**	**24/5/2019**	**26/5/2020**	**20/5/2021**	**18/10/2022**
Hydrorepellent	T9'	ΔL	0.00	−0.87	−2.80	−3.94	−8.32	−9.45	−13.55	−13.03	−18.64	^*^
		Δa	0.00	−0.07	−0.27	−0.11	−0.26	−0.27	−0.20	−0.54	−1.02	^*^
		Δb	0.00	−0.75	−0.41	−0.35	−1.62	−1.97	−2.28	−1.03	−1.33	^*^
		ΔE	0.00	1.15	2.84	3.96	8.48	9.66	13.74	13.09	18.72	^*^
	T9”	ΔL	0.00	2.85	2.86	−2.51	−3.32	−6.38	−10.83	−13.06	−14.34	^*^
		Δa	0.00	−0.13	−0.25	−0.16	−0.14	−0.25	−0.14	−0.31	−0.77	^*^
		Δb	0.00	−0.80	−0.60	−1.38	−1.52	−2.55	−2.52	−2.04	−1.03	^*^
		ΔE	0.00	2.96	2.93	2.87	3.65	6.88	11.12	13.23	14.40	^*^
	T9”'	ΔL	0.00	1.38	−0.69	−2.25	0.86	−10.80	−5.42	−8.98	−14.42	^*^
		Δa	0.00	0.07	−0.10	0.06	0.12	0.05	−0.06	−0.43	−0.89	^*^
		Δb	0.00	−0.02	0.29	0.83	1.06	−0.62	0.02	−0.02	0.71	^*^
		ΔE	0.00	1.38	0.76	2.40	1.37	10.82	5.42	8.99	14.46	^*^
	T10'	ΔL	0.00	−1.02	−3.70	−6.95	−7.89	−7.46	−8.17	−23.34	−26.44	^*^
		Δa	0.00	0.12	−0.02	0.31	0.33	0.18	0.09	−0.08	−0.65	^*^
		Δb	0.00	0.10	0.29	0.37	−0.37	−0.48	−0.26	−0.62	−0.38	^*^
		ΔE	0.00	1.03	3.71	6.97	7.91	7.48	8.17	23.34	26.45	^*^
	T10”	ΔL	0.00	0.20	−3.33	−7.72	−11.78	−12.38	−8.69	−6.65	−19.25	^*^
		Δa	0.00	0.13	−0.02	0.05	0.03	−0.12	−0.11	−0.30	−0.54	^*^
		Δb	0.00	−0.03	−0.50	−1.45	−1.73	−2.01	−2.04	0.16	0.52	^*^
		ΔE	0.00	0.24	3.37	7.86	11.91	12.54	8.93	6.66	19.27	^*^
	T10”'	ΔL	0.00	1.15	−1.17	−7.40	−8.89	−14.01	−14.95	−15.43	−13.29	^*^
		Δa	0.00	0.01	−0.17	−0.04	−0.01	−0.03	−0.07	−0.39	−0.98	^*^
		Δb	0.00	−0.50	−0.35	−1.17	−1.28	−1.72	−1.71	−0.52	−0.65	^*^
		ΔE	0.00	1.25	1.23	7.49	8.98	14.12	15.05	15.44	13.34	^*^
Control test	C	ΔL	0.00	−0.63	−3.35	−5.10	−7.13	−10.87	−16.22	−20.48	−25.14	−26.36
		Δa	0.00	0.14	−0.06	−0.11	−0.25	−0.33	−0.46	−1.19	−1.33	−1.41
		Δb	0.00	−0.26	−0.36	−1.26	−1.65	−2.60	−2.56	−1.42	−0.82	−1.33
		ΔE	0.00	0.69	3.37	5.26	7.33	11.19	16.43	20.56	25.19	26.43

^*^Indicates the area where it was not possible to distinguish the test area.

**Table 5:** L^*^ average values (in bold) and relative standard deviations (SD) recorded for each treated test area.

**L** ^*^ **values**		**03/04/2014**		**09/05/2016**		**30/05/2017**		**22/05/2018**		**24/05/2019**		**26/05/2020**		**20/05/2021**	
		**Average**	**SD**	**Average**	**SD**	**Average**	**SD**	**Average**	**SD**	**Average**	**SD**	**Average**	**SD**	**Average**	**SD**
**T1**	**T1'**	**68.97**	2.32	**70.61**	1.06	**70.12**	3.43	**67.24**	2.77	**0.00**	0.00	**56.44**	3.42	**44.89**	7.51
	**T1”**	**65.88**	1.48	**64.73**	4.47	**65.33**	5.85	**65.27**	2.54	**57.94**	4.76	**63.27**	3.15	**55.11**	5.80
	**T1”'**	**75.38**	2.67	**79.55**	2.16	**77.30**	3.70	**74.55**	5.30	**67.61**	0.40	**68.75**	6.33	**55.68**	8.13
**T2**	**T2'**	**66.30**	3.06	**68.89**	3.46	**63.28**	4.04	**64.60**	4.66	**57.30**	8.82	**53.25**	3.73	**44.76**	3.45
	**T2”**	**68.11**	1.03	**67.67**	1.92	**62.79**	2.57	**60.64**	4.92	**56.27**	2.09	**54.53**	7.18	**46.90**	7.29
	**T2”'**	**69.90**	2.42	**68.75**	3.74	**65.41**	4.49	**67.73**	3.42	**53.17**	9.26	**52.80**	7.60	**0.00**	0.00
**T3**	**T3'**	**65.10**	2.20	**66.26**	2.15	**65.76**	3.38	**66.66**	3.51	**0.00**	4.23	**62.48**	5.33	**57.00**	6.25
	**T3”**	**65.62**	0.79	**67.15**	3.09	**69.57**	3.44	**67.89**	3.03	**64.95**	3.09	**58.74**	4.43	**54.60**	5.00
	**T3”'**	**71.27**	3.53	**76.11**	3.31	**74.64**	4.58	**77.01**	3.72	**70.41**	8.91	**61.46**	7.69	**64.18**	8.82
**T4**	**T4'**	**64.92**	1.06	**65.25**	1.97	**65.03**	3.97	**67.23**	3.10	**60.99**	4.46	**61.60**	8.77	**59.02**	7.52
	**T4”**	**75.09**	3.33	**75.02**	4.03	**73.79**	5.53	**71.95**	3.67	**68.38**	6.59	**61.41**	4.16	**52.24**	6.77
	**T4”'**	**73.58**	2.07	**73.52**	2.64	**70.42**	4.99	**72.15**	2.89	**65.47**	8.45	**66.85**	5.03	**54.40**	9.66
**T5**	**T5'**	**65.27**	4.87	**61.38**	3.40	**59.16**	3.56	**57.17**	3.80	**44.99**	5.63	**42.55**	5.29	**38.26**	3.45
	**T5”**	**61.27**	2.83	**58.38**	3.76	**57.15**	5.61	**54.32**	5.92	**50.28**	4.70	**47.81**	3.38	**41.78**	4.70
	**T5”'**	**61.22**	3.93	**59.30**	3.38	**56.81**	5.05	**51.55**	4.02	**47.85**	5.82	**40.80**	4.35	**37.24**	2.70
**T6**	**T6'**	**61.35**	0.72	**60.74**	3.02	**55.00**	3.95	**53.84**	3.34	**0.00**	0.00	**44.70**	5.01	**35.56**	2.06
	**T6”**	**69.53**	1.27	**65.49**	3.66	**60.82**	4.89	**58.95**	8.32	**0.00**	5.63	**45.54**	4.99	**0.00**	0.00
	**T6”'**	**60.40**	3.32	**54.60**	3.02	**54.08**	2.29	**48.74**	3.59	**0.00**	3.57	**42.50**	4.30	**39.96**	5.31
**T7**	**T7'**	**54.56**	2.44	**53.61**	3.13	**49.60**	4.02	**47.01**	3.82	**43.84**	4.38	**41.87**	3.25	**39.32**	2.44
	**T7”**	**60.78**	2.42	**58.93**	2.19	**56.28**	3.50	**56.35**	3.23	**50.66**	4.13	**49.66**	4.37	**46.31**	3.91
	**T7”'**	**60.23**	3.57	**61.09**	4.53	**55.37**	6.19	**58.89**	8.14	**51.32**	8.13	**49.08**	5.11	**43.85**	4.36
**T8**	**T8'**	**61.26**	2.52	**63.39**	2.76	**59.98**	3.66	**59.31**	4.60	**54.40**	3.87	**48.28**	3.73	**41.90**	4.72
	**T8”**	**63.59**	2.80	**59.50**	2.93	**56.50**	4.14	**51.38**	5.46	**46.94**	3.40	**39.15**	1.42	**42.18**	6.51
	**T8”'**	**57.29**	1.59	**54.53**	2.95	**52.79**	2.33	**52.16**	4.18	**46.79**	4.68	**44.10**	2.96	**39.92**	8.38
**T9**	**T9'**	**56.76**	4.96	**52.82**	3.95	**48.44**	5.48	**47.31**	5.09	**43.21**	5.28	**43.96**	5.65	**38.12**	5.05
	**T9”**	**48.28**	2.03	**45.77**	2.33	**44.96**	4.37	**41.90**	2.77	**37.45**	3.81	**35.09**	1.99	**33.94**	1.31
	**T9”'**	**55.44**	1.69	**53.19**	5.29	**56.30**	5.72	**0.00**	0.00	**50.02**	7.20	**46.66**	6.24	**41.02**	5.81
**T10**	**T10'**	**60.78**	2.08	**53.83**	5.54	**52.89**	2.45	**53.32**	4.04	**52.61**	3.69	**37.45**	5.23	**34.34**	4.02
	**T10”**	**54.39**	1.78	**46.67**	2.74	**42.61**	4.42	**42.01**	4.45	**45.70**	5.99	**47.74**	4.41	**35.14**	1.83
	**T10”'**	**52.01**	2.49	**44.61**	4.79	**43.12**	4.25	**38.00**	2.99	**37.06**	3.05	**36.58**	3.52	**38.72**	1.21

## 4. Discussion

The life of a work of art involving live microorganisms has a duration linked to the survival or recolonization capacity of the organisms. In our case, we aimed to reduce the natural recolonization, depending on the velocity and rate of the natural colonization of the microorganisms involved. Our previous study on the Lungotevere embankments in Rome, where the Kentridge artworks were realized in 2016 (Bartoli et al., 2021a), concerning the time of natural recolonization of such microorganisms showed that during the first 2 years, no evident visual changes occurred (ΔE ^*^ < 4), but in the following 3 years, a complete regrowth was detected, with visible seasonal variations linked to rain and temperature seasonal trends (Caneva et al., 1995; Bellinzoni et al., 2003; Traversetti et al., 2018). As stated by Liebig and Shelford's laws (Odum et al., 1971) and highlighted by several authors (Caneva et al., 2008, 2015, 2016; Bartoli et al., 2014; Liu et al., 2018; Gaylarde, 2020) water was the main limiting factor controlling and conditioning the presence and the growth of these epilithic micro-communities.

Our research highlighted how using specific products can extend the artwork's life when reapplying the biocides after the fourth year. Moreover, our data also confirmed, as known, that the water repellents applied alone did not stop the microbial colonization, while water repellents plus biocides can better prevent microbial growth. However, in the case of artistic applications, such as obtaining shades of gray, the Silo 111 has shown a minimal effect in preventing biological growth and as such could be useful for the purpose.

Our results showed that applying different mixtures of the selected biocides delayed the recolonization until 2019 (after 5 years from the first application), as shown by the colorimetric data that registered variations not perceptible to the naked eye (ΔE ^*^ < 4). On the other hand, the control values on the untreated test and the data acquired in our previous study (Bartoli et al., 2021a) showed a restart of colonization [variations perceptible to the naked eye (ΔE ^*^> 4)] starting from the 2nd year. Therefore, applying the selected products delayed the recolonization process by 3 years. Since 2019, the products have not entirely inhibited biological growth but slowed them down in comparison with the control data, making the work still visible, if not as neat as in the 1st years. Most test areas in this study, after 5 years (2019), began to show variations perceptible to the naked eye (ΔE ^*^> 4).

The pre-treatment with Preventol R80^®^ was efficient over time only when combined with an application of a mixture of biocide and water repellents. In fact, for the test areas without the pre-treatment of Preventol R80^®^, it was impossible to register the colorimetric data in October 2022 because the biological patina covered the surface. In detail, the solution of Preventol R80^®^ e Biotin R^®^ plus Silo 111^®^ at both concentrations gave the best results. After 7 years, despite the restart of recolonization (ΔE ^*^> 4), the data were still lower than those recorded on the control tests for the natural recolonization. The treatment slowed the regrowth for over 2 years (Bartoli et al., 2021a). The partial efficacy over time of the biocides and water repellent may be linked to their leaching from the surface during rain events (Wittmer et al., 2011), and could influence their eco-sustainability (Bollmann et al., 2017). In recent years, there have been technological advancements in the design of biocide products used to remove biofilms on monument surfaces. It would be useful to support and extend the life of the bio-artworks, testing innovative natural biocides or natural active compounds associated with nanotechnology (Fidanza and Caneva, 2019; Palla, 2020; Ruggiero et al., 2020; Tortora et al., 2020). This could allow an eco-friendly, sustainable, and safe approach for a long-lasting artistic production.

This study acquired data over a long period in natural conditions, obtaining a realistic trend of the results. However, it is important to stress some challenges encountered when monitoring in the field, linked to the highly heterogenous substratum as the travertine, the fluctuation over the time of the microclimatic parameters, and the exposure to the public. In this way, collaboration between artists and scientists is very important to obtain a more durable and functional realization of the artwork, which is however still destined to change and disappear over time.

## 5. Conclusions

This research provides new data about the efficiency of different mixes of products in slowing down biological recolonization, with the final purpose of supporting the realization of artwork made from natural and living materials. The relevant aspect of this study is that the monitoring lasted for 8 years (October 2022) in natural conditions, showing the performance of the applied products and the natural recolonization of travertine stone. Knowing how the different products can delay biological colonization allows a longer duration for such artworks. The analysis showed that the application of the tested products slowed down the restart of colonization by 3 years compared to natural conditions. The treatment that slowed down the total recolonization and therefore allowed a longer duration of the artwork (after 7 years, the recolonization was not yet completed) consisted of a Preventol-based pre-treatment followed by an application of a mixture of the solution of Biotin R^®^ plus Silo 111^®^.

## Data availability statement

The original contributions presented in the study are included in the article/supplementary material, further inquiries can be directed to the corresponding author.

## Author contributions

FB and AC contributed to conception and design of the study, organized the database, performed the statistical analysis, and wrote the first draft of the manuscript. DI and AK organized the database and revised the first draft of the manuscript. GC contributed to conception and design of the study and revised the first draft of the manuscript. All authors contributed to manuscript revision, read, and approved the submitted version.
